# Evaluating Bias in Self-Reported Symptoms During a Cyanobacterial Algal Bloom

**DOI:** 10.3390/toxins17060287

**Published:** 2025-06-06

**Authors:** John S. Reif, Rebecca Koszalinski, Malcolm M. McFarland, Michael L. Parsons, Rachael Schinbeckler, Judyta Kociolek, Alex Rockenstyre, Adam M. Schaefer

**Affiliations:** 1 Department of Environmental and Radiological Health Sciences, Colorado School of Public Health, Colorado State University, Fort Collins, CO 80523, USA; 2 College of Nursing, University of Central Florida, Orlando, FL 32826, USA; rebecca.koszalinski@ucf.edu (R.K.); alex.rockenstyre@ucf.edu (A.R.); 3 Harbor Branch Oceanographic Institute, Florida Atlantic University, Fort Pierce, FL 34946, USA; mmcfarland@fau.edu; 4 The Water School, Florida Gulf Coast University, Fort Myers, FL 33965, USA; mparsons@fgcu.edu (M.L.P.); raschinbeckler@fgcu.edu (R.S.); 5 Clinical Research Unit, Florida Atlantic University, Boca Raton, FL 33431, USA; jkociolek2017@health.fau.edu; 6 Independent Researcher, Richmond Hill, GA 31324, USA; schaefer.a@gmail.com

**Keywords:** harmful algal blooms (HABs), cyanobacteria, environmental epidemiology, information bias, self-reported symptoms, microcystin

## Abstract

Algal blooms produced by cyanobacteria liberate microcystins and other toxins that create a public health hazard. During the 2018 bloom of *Microcystis aeruginosa* in Florida, USA, residential and recreational exposures were associated with an increased risk of self-reporting respiratory, gastrointestinal, or ocular symptoms for 125 participants. Subsequently, 207 persons were interviewed between 2019 and 2024 in the absence of large-scale algal blooms and were considered non-exposed. Analyses of cyanotoxins and brevetoxins in water and air showed only intermittent, background levels of toxins during the non-bloom period. The purpose of this report was to compare symptom reporting between active bloom and non-bloom periods. The assessment of information bias from self-reported symptoms is an important issue in epidemiologic studies of harmful algal blooms. During the non-bloom period, no statistically significant associations with residential, recreational, or occupational exposures were found for any symptom group. Estimated risks for respiratory, gastrointestinal, and ocular symptoms, headache, and skin rash were significantly higher for persons sampled during the bloom than the non-bloom period with odds ratios (ORs) of 2.3 to 8.3. ORs for specific respiratory symptoms were also significantly elevated. After adjustment for confounders and multiple exposures in multivariable analyses, the differences in symptom reporting between bloom and non-bloom periods remained statistically significant. In summary, the use of self-reported symptoms in this epidemiologic study of exposure to a cyanobacterial algal bloom did not appear to introduce substantial information bias.

## 1. Introduction

The population of Florida, USA, has been exposed historically to recurring harmful algal blooms (HABs) caused by two classes of organisms. Blooms of blue-green algae (cyanobacteria) have become more prevalent in Florida and worldwide due to factors including climatic changes and agricultural practices. Warming ocean temperatures and heavy nutrient loading, particularly with phosphates, have exacerbated the frequency and intensity of cyanobacterial blooms in Florida and elsewhere [1,2,3,4]. Large-scale blooms of *Microcystis aeruginosa* occurred in Florida during the summers of 2016 and 2018 [2]. Both blooms originated in Lake Okeechobee, the largest lake in the southeastern United States. Heavy rainfalls and rising lake heights prompted the U.S. Army Corps of Engineers to release *Microcystis*-contaminated water from the lake to protect human safety [2]. These discharges traveled downstream via a human-made canal system to the St. Lucie Estuary and Indian River Lagoon to the east and the Caloosahatchee River to the west coast [5]. The Florida Department of Environmental Protection [6] detected blue-green algae with high concentrations of microcystins (MCs) at multiple sites exceeding the U.S. EPA standard for recreational waters (8.0 ppb) protective against acute health effects [7]. The contamination of local waterways created a public health concern for adjacent communities resulting in the State of Florida declaring Public Health Emergency for the 2016 event [4].

Cyanobacteria produce a suite of toxins including the hepatoxic microcystins, (MCs), and nodularins, as well as cylindrospermopsins, anatoxins and β-N-methylamino-L-alanine (BMAA), with implications for human health [2]. The microcystins are hepatotoxic cyclic heptapeptides with over 270 known congeners, among which microcystin-LR is the most toxic form [8]. Human exposure to MCs may occur by the inhalation of aerosols, consumption of contaminated water or seafood, or direct contact with the skin [9]. The primary health effects known to be induced through these exposures include irritative symptoms of the upper and lower respiratory tract and eyes, gastrointestinal symptoms including vomiting and diarrhea, and skin rash [10].

The scientific literature concerning health effects attributable to exposures to cyanotoxins is relatively sparse. Important data gaps exist for chronic effects that may occur after exposure that would be best answered in prospective cohort studies. In particular, cohort studies should address the long-term effects that may follow acute hepatoxicity [11], albeit at lower doses. Additional information concerning the roles of endotoxins and allergy would be useful to enhance understanding of the pathogenesis of reported health effects. In vivo studies of the roles of cytokines and other immune mediators in laboratory animals and humans are needed [12]. The potential role of exposure to cyanotoxins such as β-Methylamino-L-alanine or BMAA on neurodegenerative disorders is under active investigation [13].

The proliferation of the toxic marine dinoflagellate *Karenia brevis* produces a suite of highly irritative brevetoxins found in Florida red tides [2]. Red tides occur primarily in the Gulf of Mexico along the western coast of the state and are most likely to occur during late summer and fall. Rapidly increasing population growth, particularly in coastal areas, eutrophication, increases in hurricane severity, and increases in water temperature have exacerbated risks to human health [2]. Red tide health effects in humans are due primarily to the inhalation of aerosol-containing brevetoxins, which result in an acute inflammatory response and a well-characterized syndrome of respiratory tract symptoms [14]. Aerosolized exposure has been reported to cause acute respiratory symptoms, including nonproductive cough, wheezing, chest tightness, shortness of breath, and eye irritation [15]. Environmental exposure to brevetoxins in Florida caused a significant increase in reported symptoms and a decline in pulmonary function in a cohort of people with pre-existing asthma [16]. The consumption of brevetoxin-contaminated shellfish (clams, mussels, and oysters) is another, but less common route of human exposure resulting in gastrointestinal symptoms [2].

In a study of 125 persons potentially exposed to cyanobacterial toxins during the 2018 bloom in Florida, MCs were detected in nasal swabs from 95% of the participants sampled [17]. The inhalation of aerosols was suggested to be an important pathway of exposure during this large-scale bloom event. MCs were detected in the nasal passages of persons who reported being in proximity to the bloom but denied direct contact with impacted waters.

A second investigation was conducted to characterize health effects associated with the 2018 bloom of blue-green algae [1]. Analyses were conducted to determine whether self-reported symptoms were associated with activity patterns, direct contact with water, residential, recreational, or occupational exposure. Symptoms reported commonly were rhinorrhea, sneezing, headache, sore throat, and dry cough. Respiratory symptoms were reported by 74%, ocular symptoms by 62%, and gastrointestinal symptoms by 35% of respondents. Residential and recreational exposures were significantly associated with increased risks of respiratory, gastrointestinal, and ocular symptoms [1].

A methodologic concern in prospective or cross-sectional epidemiologic studies based on self-reported symptoms is the possibility that over-reporting may lead to information bias. Persons who consider themselves exposed or are concerned about the health effects of exposure may report symptoms more frequently. This form of recall bias may lead to differential misclassification of outcomes with a bias away from the null [18]. Our previous study did not include a concurrent non-exposed comparison group [1]. Therefore, the current investigation was conducted (a) to determine whether significant associations with residential, recreational, or occupational exposures to local water bodies during non-bloom years of 2019 to 2024 existed; and (b) to compare the responses of persons interviewed during the non-bloom years of 2019 to 2024 with those of persons interviewed during the large-scale bloom of 2018. Collectively, these analyses permit an indirect assessment of the degree to which reporting bias may have affected the results obtained during the 2018 bloom.

## 2. Results

The cyanobacterial blooms that occurred in central Florida in 2016 and 2018 are in clear contrast to background levels of microcystins and other cyanotoxins that occur intermittently throughout the year. These extensive or “large scale” blooms persisted for several months and were widely distributed geographically. The concentrations of microcystins exceeded regulatory thresholds, and in the case of the 2016 bloom, caused the state of Florida to declare a Public Health Emergency [4,5]. Sporadic, intermittent blooms of cyanobacteria and *Karenia* of limited intensity, geographic distribution, and duration are common in Florida [2] due to favorable environmental conditions and are monitored by several state agencies. The results of investigator sampling for HAB toxins in water and air in the non-bloom years of 2019 to 2024 are described below.

### 2.1. Environmental Toxin Data—Water Concentrations

Between 2019 and 2024, sporadic blue-green algal bloom activity was detected in water. A total of 739 water samples were obtained from the St. Lucie Estuary and Lake Okeechobee. Microcystin was detected in 21.2% of these samples; 5.27% of samples had concentrations above the U.S. EPA recreational exposure limit of 8 ppb [7]. Overall, these data show some cyanobacterial activity at selected locations. For example, the highest microcystin concentrations recorded were found in Lake Okeechobee during June 2020. Blooms of blue-green algae, determined to be *Microcystis aeruginosa,* were evident on the lake surface. Microcystin concentrations ranged from below detectable limits to >5000 ppb. However, bloom activity was restricted to the southern part of the lake and lake water was not discharged downstream in substantial amounts. Samples collected to the east (St. Lucie Estuary) and west (Caloosahatchee River Estuary) of Lake Okeechobee had low or undetectable concentrations of microcystins, presumably due to limited freshwater releases from the lake. None of these sampling events revealed extensive cyanobacterial bloom activity comparable to that observed in 2016 and 2018. Similarly, quantifiable brevetoxin concentrations were found during October of 2019 near the mouth of the Caloosahatchee River Estuary and during July 2020. No active blooms of *Karenia brevis* were observed during sample collection at these sites.

### 2.2. Environmental Toxin Data—Air Concentrations

The total microcystin loading (i.e., the sum of the loadings across all of the air filters within each impactor) ranged from 0.0006 to 2.386 ng m^−3^. The upper value was measured at Lake Okeechobee on 6 December 2022. Toxin loadings on the Stage 6 and 7 filters (i.e., those that capture particles < 1 µm, most likely to reach the small bronchioles and alveoli within the lung) were 5-fold lower than the total loading in each sampler, <0.0001–0.5489 ng m^−3^ (averaging 0.0352 ng m^−3^). Similar concentrations were measured over the course of the study, showing that low concentrations of microcystins are present in ambient air during non- (or low-) bloom sampling scenarios.

Limited evidence of brevetoxin in air samples was found. Two of the sixty-two filters analyzed from Cape Coral in February 2023 contained detectable concentrations of brevetoxin when a red tide was active off-shore in the Gulf of Mexico.

### 2.3. Statistical Analyses

#### 2.3.1. Demographic Characteristics

Demographic characteristics and exposure patterns were compared between the 125 persons sampled during the 2018 bloom [1] and the 207 persons sampled in the non-bloom period of 2019 to 2024 (Table 1). Statistically significant differences were found for several variables. Those sampled during the bloom period were younger, and more likely to be male and to be Caucasian. There were no significant differences in educational achievement, a measure of socio-economic status. Residential exposure was reported more significantly more frequently during the non-bloom period, whereas recreational and occupational exposure and direct contact with water were more common among participants sampled during the 2018 bloom (*p* < 0.05).

There were no statistically significant differences reported between groups with respect to a history of asthma or chronic pulmonary disease. Persons sampled during the bloom period were more likely to have a history of hay fever or seasonal allergy (*p* < 0.001).

#### 2.3.2. Analysis of Symptom Data for Non-Bloom Participants

The analysis of symptom data is shown in Table 2 for non-bloom participants. None of the symptom groups showed a statistically significant difference in reporting for either residential or recreational exposure, in contrast to what was reported for the 2018 bloom [1]. There were also no significant differences in symptom reporting for persons with and without occupational exposure. Eleven persons reported eating locally caught fish in the past 10 days; one reported gastrointestinal symptoms.

Responses to the question “Are you concerned about having contact with blue-green or red tide algae and possible effects on your health?” were analyzed in the 2019 to 2024 data. Concern about health effects was common, with 68.1% responding affirmatively. Those expressing concern were older than those who did not (mean 58.8 ± 13.5 vs. 51.4 ± 15.8 years, *p* = 0.04). There was no significant difference in concern between men and women. Caucasians were more likely to have answered “yes” to this question than other racial groups. Residential exposure was the main risk factor for concern about health effects and exposure to HABs (*p* < 0.001) Concerned persons were more likely to have lived on a waterway (76.1 vs. 33.3%, *p* < 0.001) and more likely to report a respiratory symptom (51.0% vs. 16.7%, *p* < 0.001) during the non-bloom period. None of the other outcomes were reported significantly more frequently by those who expressed concern about exposure to HABs.

#### 2.3.3. Comparison of Symptom Data from Bloom and Non-Bloom Participants

In univariate analyses, the frequencies for all major symptom groups were significantly higher for the 2018 bloom participants than for those from the non-bloom period (Table 3). ORs ranged from 2.27 for sneezing to 6.56 for fever. The estimated risks for individual respiratory symptoms ranged from 2.3 to 8.25 during the bloom (*p* < 0.001). The higher prevalence of hay fever or seasonal allergy reported by bloom participants could have increased reporting for ocular symptoms, runny nose and sneezing. However, the risk estimates for these outcomes during the bloom were of the same order of magnitude as those for other symptoms not part of the seasonal allergy complex.

Multivariable logistic regression was used to control for potential confounding and multiple pathways of exposure (Table 4.) Variables that were significantly different in the univariate analyses: age, sex, race, residential, recreational, and occupational exposure, were included in the forward selection modelling for each symptom group and specific symptom. Age, sex, and residential, recreational, and occupational exposure were retained in the final models for all symptom groups. Residential exposure, race, and sex did not meet the criterion for retention for wheezing and headache. The frequencies of reporting for all major symptom groups remained significantly higher for the bloom period after adjustment for covariates. Adjusted ORs were similar to those reported in the univariate analyses. A separate model with direct contact with water in the past 24 h forced into the model was used to estimate risk for skin rash. An odds ratio of 6.72 was calculated but the estimate was highly imprecise (95% CI 1.95–29.22) due to the low prevalence of reported rash in the total sample (n = 21).

## 3. Discussion

The literature concerning the health effects of exposure to cyanotoxins is based largely on case reports and anecdotal evidence [19]. In contrast, there have been a limited number of formal epidemiologic studies conducted to date. Most used a cross-sectional design in which exposure and self-reported symptoms were determined at the same time. The validation of symptoms by a medical provider was not conducted, with one exception [20]. Backer et al. used a comparison population of unexposed persons to improve study validity [9]. The remaining cross-sectional studies did not include a non-exposed comparison group.

A limited number of prospective cohort studies have been conducted to assess health effects associated with exposures to cyanobacterial blooms. In cohort designs, individuals are disease-free at enrollment and followed to measure incidence of new events, thus limiting the potential for recall bias. An increased risk of symptoms two to seven days after recreational exposure to >5000 cyanobacterial cells/mL was reported for 852 cohort members in Australia [21]. Stewart et al. [22] assessed symptoms prospectively by phone interview three days after contact with water for over 3,500 people at multiple sites. A two-fold increase in the risk of respiratory symptoms for boating on lakes with higher levels of cyanobacteria (cell surface area > 12.0 mm^2^/mL) compared to lakes with lower levels of cyanobacteria (<2.4 mm^2^/mL) was reported. A prospective study of beach-goers in Puerto Rico showed significant associations between phytoplankton cell counts and self-reported eye irritation, respiratory illness, and rash 10 to 12 days after the beach visit [23]. The strongest and most consistent associations were found for cyanobacteria. In a Canadian cohort study in which participants kept a daily log, an increased risk of gastrointestinal symptoms was associated with residence on lakes containing > 100,000 cyanobacterial cells/mL [24]. Although the quality of symptom reporting was improved in the prospective studies, the responses were self-reported. The cohort study in the current analysis [1] had limited follow-up of individuals to assess potential long-term effects of HAB exposure on participants.

Several forms of potential bias should be considered in evaluating these results. First, the associations described in the analysis of persons exposed to the cyanobacterial bloom of 2018 [1] do not appear to be due to self-reporting bias. The frequencies of symptom reporting for all major outcomes during the 2018 bloom were significantly higher than the frequencies for the non-bloom period of 2019–2024 after adjustment for potential confounding and multiple exposure pathways. Conversely, if reporting bias had been responsible for the differences seen in the 2018 data, the results from the non-bloom period would have more closely resembled those from the bloom period.

Second, the possibility of exposure misclassification should be considered in comparing symptom reporting between bloom and non-bloom periods. As shown above, there was intermittent low-level activity of cyanobacteria and *Karenia brevis* at selected locations and times during the non-bloom period. These sporadic episodes could have led to limited human exposure to toxins. The effect of such exposures during the non-bloom period would have been to cause exposure misclassification. If persons presumed to be non-exposed are in fact exposed, the result would be to decrease the magnitude of the effect estimates (bias towards the null) in comparing bloom and non-bloom participants [18]. Despite the potential for limited exposure misclassification, the risk estimates for all outcomes are significantly higher for persons sampled during the 2018 bloom.

Third, persons who perceive a health risk from an exposure may over-report symptoms. This form of information bias was studied in a series of randomized trials with bathers blinded to their individual exposures to polluted marine recreational waters [25]. Self-reported skin disorders were subject to “risk perception bias”, as shown by the reduction in crude risk after adjustment for subjects’ perceived risk [25]. The 2019-to-2024 questionnaire incorporated a question designed to assess concern about health risks and exposure to HABs to address this issue. This question was not included in the original questionnaire used in 2018. Therefore, a direct comparison of concern could not be included in the multivariable models of inter-period differences in symptom reporting. Incorporation of residential exposure in the models may have mitigated this deficiency since those individuals who lived on waterways were more likely to have reported health concerns about exposure to HABs. The over-reporting of respiratory symptoms by persons who were concerned during the non-bloom period would be expected to have reduced the magnitude of the difference for this outcome between reporting periods. As shown above, the difference for all respiratory symptoms between reporting periods was five-fold and in the range of 2.3 to 8.1 for individual respiratory symptoms.

The strong associations with respiratory outcomes shown here reinforce the importance of aerosol exposures in the pathogenesis of airway disease following exposure to microcystins. The symptom profiles suggest an inflammatory response potentially mediated by a cytokine response, as supported by the up-regulation of pro-inflammatory genes after the administration of microcystin-LR on a primary human airway epithelial cell line [26]. Polymerase chain reaction analyses of fluid obtained by bronchoscopic lavage showed cyanobacteria (*Microcystis*) present in the lungs of persons who did not have direct contact with a water body [27]. As described previously [1], there is good evidence that aerosol dispersion of cyanobacterial toxins occurs during HABs blooms from surface water to inland locations [28,29]. Further, the particle sizes in these bioaerosols < 3.3 μm) are of respirable dimensions, permitting them to reach the lower airways of the lung [30].

The analyses reported here were designed to determine whether information bias from self-reporting may have affected the results of the previously reported study [1] by incorporating a negative comparison group. The incorporation of negative controls in observational research increases the validity of causal inference by reducing the likelihood that confounding, recall bias, or other sources of error may have led to spurious results [31]. Our results are relevant to other epidemiologic studies of health effects and exposure to HABs, which typically rely on self-reporting in both cross-sectional and prospective cohort designs [25]. In summary, relatively few prospective studies of exposure to marine phytoplankton have been reported. The study reported here adds to the evidence that blooms of cyanobacteria constitute a public health hazard for persons recreating and living in the vicinity of impacted waterways [5].

## 4. Methods

### 4.1. Human Subjects

Beginning in 2019, participants were sampled from the east coast of Florida. These participants resided primarily in Martin and St Lucie counties, with potential exposure to blue-green algal blooms caused by *Microcystis aeruginosa*, as described previously [1,17]. Sampling sites in the vicinity of Stuart, FL with local access to the St Lucie River and Indian River Lagoon and communities on Lake Okeechobee were included. In 2020, the recruitment of participants from the west coast was initiated to expand the study to persons potentially exposed to brevetoxins liberated during red tide events. West coast participants resided primarily in Lee County and had potential exposure to brevetoxins from the Gulf of Mexico, the tidal Caloosahatchee River, and over 400 miles of canals in the Cape Coral area. Several locations on the west coast of Florida have been shown to experience both *Karenia brevis* and cyanobacterial blooms [5], indicating that residents might be exposed to either or both toxins.

The recruitment of participants followed procedures used in the previous studies [1,17]. Initial outreach consisted of the use of social media and distribution of flyers to local interest groups, businesses, and local health departments. Persons 18 years of age and older who were able to speak and read English and who lived or worked in any of the study areas on the east or west coasts of Florida were eligible for inclusion. Voluntary participation was sought by informing the public that the investigators were recruiting participants for a research study to evaluate potential short- and long-term health effects of exposure to harmful algal blooms. The Florida Atlantic University (FAU) Institutional Review Board approved all components of the study as protocol # 2310217.

After obtaining written informed consent, participants were asked to complete a questionnaire as originally modified from Backer et al. [9] and updated through several iterations following the analysis of the 2018 data. The questionnaire recorded demographic information, smoking and alcohol consumption history, residential proximity to a waterway, and recreational activities resulting in contact with specific water bodies during the prior 10-day period. Recreational activities included swimming, boating, fishing, jet skiing, paddle boarding, and spending time at a local beach with or without immersion. Direct contact with specific water bodies during the prior 24 h was also recorded. Potential occupational exposure was assessed through job title and contact history with local waterways. The consumption of locally caught fish and shellfish by species and amount in the past 10 days was ascertained. Questions concerning relevant medical history including asthma, seasonal allergy/hay fever, chronic obstructive pulmonary disease, chronic gastrointestinal disorders, and liver disease were included. A yes/no history of specific symptoms experienced by the participant during the prior 10 days was obtained. These included those affecting the respiratory system (7), the gastrointestinal system (4), and the eye (3), and headache, fever, and skin rash. Validation by medical record retrieval or physician/provider contact was not attempted. A question regarding participant’s level of concern about HABs was included: “Are you concerned about having contact with blue-green or red tide algae and possible effects on your health?”

### 4.2. Environmental Sampling—Water

Water samples were collected by the Harbor Branch Oceanographic Institute (HBOI), the Florida Department of Environmental Protection (FDEP), South Florida Water Management District (SFWMD), and various county officials to monitor bloom activity and algal toxin concentrations throughout the study period. The FDEP, SFWMD, and county personnel also collected water samples as part of their routine monitoring activity or in response to reports of bloom events. Data from samples collected by state agencies or counties were obtained through the FDEPs Division of Environmental Assessment and Restorations Algal Bloom Sampling Results website (https://floridadep.gov/AlgalBloom) and used to augment and validate the results of analyses by the investigators. The data used to define bloom and non-bloom periods for this analysis were based primarily on results of sampling by HBOI personnel.

Study personnel routinely collected water samples from Lake Okeechobee, the St. Lucie canal and estuary, and sites in and around the Caloosahatchee River Estuary near Cape Coral during the study period for analyses of microcystins and brevetoxin, phytoplankton cell counts, and chlorophyll concentrations. Additional, focused sampling of water for analysis of microcystins and brevetoxin concentrations was conducted during the week before and the day of each human sampling event at a nearby location.

Surface samples were collected in 500 mL PET or PETG bottles, stored in a cooler at ambient temperature, and returned to the laboratory within 6 h for processing. Subsamples of whole and filtered water (~15 mL each) were transferred to 20 mL glass scintillation vials and stored at −20 °C. Whole water samples were subjected to 3 freeze/thaw cycles to lyse cells prior to analysis and tested for microcystins or brevetoxins using ELISA kits provided by Abraxis LLC (Warminster, PA, USA) as previously described [17]. Assays were conducted in 96-well plates and analyzed with a plate-reading spectrophotometer at 450 nm. All samples were analyzed in duplicate. QA/QC standards included a Laboratory Reagent Blank and positive standards provided with each kit [32]. The R^2^ value for standard curves was generally >0.99. The ELISA for microcystins and nodularins is based on the detection of the AddA subunit of these molecules, required for the toxicity of the compounds. The functional lower limit of detection (LLOD) for microcystin was the higher of the measured value for the Laboratory Reagent Blank (LRB) provided with the kit or the declared kit LLOD of 0.016 µg/L. The lower limit of quantification (LLOQ) for microcystins was 0.05 µg/L, the value of the lowest positive kit standard [32]. For brevetoxin, the LLOD was 0.031 ppb as specified by the kit. Most samples were also analyzed to determine extracted chlorophyll concentrations by fluorometry by EPA method 445.0 [33], analyzed by flow cytometry to determine algal cell concentrations, and examined using light microscopy to identify dominant algal species. Additional quality control was conducted by comparing measured microcystin and brevetoxin concentrations determined by ELISA to light microscopy to confirm the presence or absence of toxic algae.

### 4.3. Environmental Sampling—Air

Andersen-style impactor Tisch TE-20-800 bulk air samplers ((Tisch Environmental, Cleves, OH, USA) were deployed in three regions (Cape Coral, Stuart, and Lake Okeechobee) between 2020 and 2024. These air samplers are designed to sample and collect airborne particulates on the different filter stages (0 to 7, and F) representing a virtual pulmonary system from the nasal tract (stage 1) to the alveolar ducts (stage 7). In total, 72 samplers were deployed and 648 Whatman 41 filters (CAT No. 1441-866) from the 9 stages were extracted and analyzed in duplicate in 22 separate sampling events. Filters were weighed and halved based on weight proportion for microcystin extraction using a Mettler Toledo scale (Mettler Toledo, Columbus, OH, USA). Filters were then submerged and homogenized in HPLC grade methanol twice, with the supernatant pipetted off. The extract was dried down in a Savant Speedvac vacuum concentrator (Thermo Fisher Scientific, Waltham, MA, USA) and reconstituted in 1 mL of methanol for ELISA analysis. Microcystin toxin concentrations were measured using Abraxis ELISA testing kits (microcystin ADDA PN 520011, Abraxis LLC, Warminster, PA, USA) with external standards and controls for QA/QC. To account and adjust for matrix effects, additional filters were spiked and extracted with predetermined toxin concentrations. Microcystin loading (ng microcystin-LR m-3) was calculated by dividing the microcystin concentrations quantified from the impactor filters by the volume of air sampled over the ~2-day deployments, where air flow was calibrated (28.3 Actual Liters Per Minute [ALPM]) through the samplers using a Gilian^®^ Gilibrator 2 calibration system (Thermo Fisher Scientific, Waltham, MA, USA). Select filters (from stages 0, 4, and F) from spring 2023 deployments in Cape Coral were also extracted and tested for brevetoxins and toxin loading was calculated as described above. A total of 62 filters were extracted and run in duplicate for brevetoxin analysis on MarBionc ELISA kits (SeaTox Research, Wilmington, NC, USA).

### 4.4. Statistical Analyses

The initial design of this study was a longitudinal prospective study in which individuals would be re-sampled during bloom and non-bloom periods. To date only limited numbers of participants have been re-sampled. Therefore, all data represent the initial interview with each participant. The 125 persons potentially exposed to MCs during the 2018 active bloom comprised the bloom period cohort members [1]. The 2019 to 2024 participants in the analyses (n = 207) represented their initial sampling event during the non-bloom period. A database was then constructed containing all 332 persons sampled between 2018 and 2024. The definitions of exposure were standardized across all the study years to account for variations across several iterations of the questionnaire. The format for demographic variables and symptom reporting was consistent across all the study years and required no modifications. The definitions of residential, recreational, and occupational exposures are described above.

Between 2019 and 2024, individuals were sampled on both the east and west coasts of Florida. The demographic characteristics of each geographic group were compared to assure comparability prior to creating a merged data set of persons sampled during the non-bloom period. No significant differences in age, sex, or level of educational achievement were found; therefore, the data for the non-bloom period were merged between east and west coast residents to create a final sample size of 207.

Demographic variables and exposure status (residential, recreational, and occupational) were compared between the populations sampled in 2018 and 2019–2024 (non-bloom period). The prevalences of co-morbid conditions such as asthma, seasonal allergies/hay fever, chronic obstructive pulmonary disease, and chronic gastrointestinal disorders were also compared between bloom and non-bloom participants. Chi Square was used to evaluate differences in categorical variables, and a *t*-test was used to compare mean ages. A *p* value of <0.05 was considered statistically significant. Fisher’s exact confidence intervals and *p* values were calculated for any comparison with a cell size < 10.

Odds ratios (ORs) with their 95% confidence intervals (CIs) were calculated to estimate risk of reporting individual symptoms and symptom groups in the non-bloom participants. The objective was to determine whether significant associations existed similar to those reported for the 2018 participants [1]. Symptom groups included any respiratory symptom, gastrointestinal, or ocular symptom, headache, and skin rash. Risks were calculated for residential and recreational exposures since these activities showed significant associations with symptoms in the earlier analysis [1]. A sub-analysis was performed to determine whether participants’ expressed level of concern about health effects from exposure to blue-green algae was associated with any of the symptom groups among persons sampled during the non-bloom period.

In the next phase of the analysis, the prevalences of specific reported symptoms and symptom groups were compared between the 2018 bloom participants and those sampled between 2019 and 2024 in the absence of a large-scale bloom of *Microcystis aeruginosa* or *Karenia brevis*. A detailed analysis of specific respiratory symptoms was performed to focus on the outcomes that showed the highest risks during the 2018 bloom [1]. Univariate analyses were conducted in Epi Info for Windows. Version 7.2. Centers for Disease Control and Prevention CDC, Atlanta, GA, USA.

Study participants may have experienced exposure to HAB toxins through multiple pathways of exposure and activities. Further, we wished to eliminate the possibility of confounding by age, sex, race/ethnicity, or socioeconomic status (education). Several significant differences in demographic characteristics and exposure profiles were identified between the 2018 and 2019–2024 population samples. Therefore, the data were further analyzed by comparing symptom reporting between the two periods using multiple logistic regression to calculate adjusted ORs and 95% CIs. Conditional forward selection iterative models were generated for each symptom group, headache, fever, and skin rash using a *p*-value of <0.10 for retention at each step of the modelling process [34]. Separate models were run for residential, recreational, and occupational exposure. The final model included age, sex, and the residential, recreational, and occupational exposure variables. Multivariable analyses were conducted in SPSS (SPSS Statistics Version 30, SPSS, Chicago, IL, USA).

## Figures and Tables

**Table 1:** Demographic Profiles and Exposure Status of Persons Sampled during Bloom and non-Bloom periods.

Participant Characteristic	Bloom Period 2018 (n = 125)	Non-Bloom Period 2019–2024 (n = 207) *	*p*-Value
Mean Age (±SD)	47.1 (14.9)	56.3 (14.6)	0.55
Sex			0.01
Male	68 (54.4)	82 (40.0)	
Female	57 (45.6)	123 (60.0)	
Race/Ethnicity			<0.02
Caucasian	119 (95.2)	173 (87.4)	
Other	6 (4.8)	25 (12.6)	
Education			0.26
High School/Some College	36 (28.8)	70 (34.5)	
College Degree/Post-Grad Education	89 (71.2)	131 (65.5)	
Residential Exposure	59 (47.2)	123 (61.5)	0.03
Recreational Exposure	63 (50.4)	79 (38.2)	0.03
Occupational Exposure	50 (40.0)	44 (21.2)	<0.001
Direct Contact With Water in Past 24 h	66 (52.8)	60 (29.0)	<0.001

* Sample sizes vary due to missing data.

**Table 2:** Prevalence of Reported Symptoms during the non-Bloom Period by Residential and Recreational Exposure, 2019–2024, Odds Ratios (OR), and 95% confidence interval (CI).

Exposure Status	Residential Exposure n = 123			Recreational Exposure n = 79		
Symptom Reported	N	OR, 95% CI	*p*-Value	N	OR, 95% CI	*p*-Value
Any Respiratory Symptom (n = 83)	54	1.48, 0.82–2.63	0.18	34	1.22 0.69–2.15	0.50
Any Gastrointestinal Symptom (n = 30)	17	0.86, 0.40–1.93	0.74	10	0.78, 0.35–1.77	0.56
Any Ocular Symptom (n = 41)	29	1.85, 0.88–3.88	0.10	12	0.61,0.21–1.28	0.19
Headache (n = 38)	19	0.63, 0.31–1.27	0.19	12	0.70, 0.33–1.49	0.36
Skin Rash (n = 8)	7	5.01, 0.62–522.4	0.15 *	3	0.97, 0.15–5.16	0.97 *

* Fisher’s Exact *p*-value and CI.

**Table 3:** Prevalence of Reported Symptoms during Bloom and non-Bloom Periods. With Estimated Risk (OR, 95% CI) for Bloom vs. Non-Bloom Period *.

Bloom Status	Bloom 2018 n = 125		Non-Bloom 2019–2024 n = 207		Odds Ratio	95% Confidence Interval	*p*-Value
Symptom Reported	N	%	N	%			
Any Respiratory Symptom	93	74.4	83	40.1	4.34	2.66–7.08	<0.001
Runny nose	63	50.4	60	29.0	2.49	1.60–3.95	<0.001
Repeated sneezing	54	43.2	52	25.1	2.27	1.41–3.64	<0.001
Productive cough (phlegm)	39	31.2	23	11.1	3.63	2.04–6.45	<0.001
Dry cough (no phlegm)	47	37.6	38	18.4	2.68	1.62–4.44	<0.001
Difficulty breathing	25	20.0	14	6.7	3.45	1.72–6.92	<0.001
Wheezy breathing	19	15.2	17	8.2	2.47	1.19–5.13	0.01
Sore throat	49	39.2	15	7.2	8.25	4.37–15.60	<0.001
Any Gastrointestinal Symptom	44	35.2	30	14.5	3.20	1.88–5.46	<0.001
Any Ocular Symptom	62	49.6	41	19.8	3.98	2.44–6.51	<0.001
Fever	11	8.8	3	2.4	6.56	1.68–37.16 *	0.003 *
Headache	54	43.2	38	14.4	3.38	2.05–5.57	<0.001
Skin Rash	13	10.4	8	3.8	2.89	1.0–7.8 *	0.02 *

* Fisher’s Exact *p* value and CI.

**Table 4:** Adjusted * Risk Estimates for Major Symptoms (Odds Ratios and 95% Confidence Intervals) for Bloom vs. non-Bloom Periods from Multiple Logistic Regression Models.

Symptom Reported	Odds Ratio	95% Confidence Interval	*p*-Value
Any Respiratory Symptom	5.34	2.96–9.63	<0.001
Runny nose	2.99	1.74–5.17	<0.001
Repeated sneezing	2.35	1.35–4.06	0.002
Sore throat	8.11	3.90–16.86	<0.001
Productive cough (phlegm)	4.58	2.34–8.95	<0.001
Dry cough (no phlegm)	3.33	1.84–6.04	<0.001
Difficulty breathing	3.50	1.54–7.95	0.003
Wheezy breathing	4.71	2.26–9.83	<0.001
Any Gastrointestinal Symptom	2.55	1.38–4.69	0.003
Any Ocular Symptom	4.21	2.34–7.57	<0.001
Fever	5.89	1.45–23.89	0.013
Headache	3.73	2.08–6.67	<0.001
Skin Rash	3.01	1.05–8.59	0.04

* Adjusted for age, sex, race, residential, recreational, and occupational exposure in the final multivariable model except as noted.

## Data Availability

The original contributions presented in this study are included in this article. Further inquiries can be directed to the corresponding author.
